# Depletion of Alveolar Macrophages Ameliorates Virus-Induced Disease following a Pulmonary Coronavirus Infection

**DOI:** 10.1371/journal.pone.0090720

**Published:** 2014-03-07

**Authors:** Stacey M. Hartwig, Kaitlyn M. Holman, Steven M. Varga

**Affiliations:** 1 Department of Microbiology, University of Iowa, Iowa City, Iowa, United States of America; 2 Interdisciplinary Graduate Program in Immunology, University of Iowa, Iowa City, Iowa, United States of America; 3 Department of Pathology, University of Iowa, Iowa City, Iowa, United States of America; MRC National Institute for Medical Research, United Kingdom

## Abstract

Coronaviruses cause respiratory disease in humans that can range from mild to severe. However, the pathogenesis of pulmonary coronavirus infections is poorly understood. Mouse hepatitis virus type 1 (MHV-1) is a group 2 coronavirus capable of causing severe morbidity and mortality in highly susceptible C3H/HeJ mice. We have previously shown that both CD4 and CD8 T cells play a critical role in mediating MHV-1-induced disease. Here we evaluated the role of alveolar macrophages (AM) in modulating the adaptive immune response and subsequent disease. Depletion of AM using clodronate liposomes administered prior to MHV-1 infection was associated with a significant amelioration of MHV-1-induced morbidity and mortality. AM depletion resulted in a decreased number of virus-specific CD4 T cells in the lung airways. In addition, a significant increase in the frequency and total number of Tregs in the lung tissue and lung airways was observed following MHV-1 infection in mice depleted of AM. Our results indicate that AM play a critical role in modulating MHV-1-induced morbidity and mortality.

## Introduction

Coronaviruses (CoV) such as 229E, OC43, NL63 and HKU1 have been shown to cause mild acute respiratory disease in humans [1]–[5]. Severe acute respiratory syndrome (SARS) is caused by a group 2 CoV termed SARS-CoV that emerged from China in late 2002 [6]–[13]. Although most CoV do not induce respiratory disease in mice, intranasal infection with the group 2 CoV mouse hepatitis virus type 1 (MHV-1), results in a pulmonary infection in mice [14].

We have previously shown that intranasal MHV-1 infection of C3H/HeJ mice, which harbor a natural mutation in the gene that encodes toll-like receptor 4 (TLR4) [15], [16], results in increased morbidity and mortality along with severe pulmonary disease as compared to the wild-type C3H/HeN mice [17]. Both CD4 and CD8 T cells contribute to the MHV-1-induced disease, as depletion of either subset ameliorates morbidity and mortality [18].

Here we assessed the role of alveolar macrophages (AM) in the induction of the pathogenic T cell response following intranasal MHV-1 infection in susceptible C3H/HeJ mice. Administration of clodronate liposomes (CL) prior to MHV-1 infection resulted in the depletion of AM within the lung. We demonstrate that mice treated with CL exhibit a significant decrease in morbidity and mortality following MHV-1 infection as compared to control mice. In addition, amelioration of MHV-1-induced disease correlated with a significant increase in the frequency and total number of Foxp3^+^ Tregs in the lung airways and the lung tissue. Our data indicate that AM play a critical role in controlling MHV-1-induced disease severity.

## Methods

### Ethics Statement

All experimental procedures utilizing mice were approved by the University of Iowa Animal Care and Use Committee. The experiments were performed under strict accordance to the Office of Laboratory Animal Welfare guidelines and the PHS Policy on Humane Care and Use of Laboratory Animals.

### Animals

Female C3H/HeJCr and C3H/HeNCr mice were purchased from the National Cancer Institute (NCI, Fredrick, MD) and were used at 5 to 8 weeks of age. All animals were maintained in accredited facilities at the University of Iowa (Iowa City, IA).

### Virus growth and infection

MHV-1 was obtained from the American Type Culture Collection (ATCC Manassas, VA) and propagated in DBT cells as previously described [18]. Mice were infected intranasally (i.n.) with 5×10^3^ plaque forming units (PFU) of MHV-1 in a total volume of 50 µl while under light anesthesia with isoflurane. Each mouse was monitored daily for weight loss and severity of illness, using a previously described scoring system [19].

### Measurement of airway function

Airway function was measured using a whole-body plethysmograph (Buxco Electronics, Wilmington, NC) as previously described [18]. Conscious, unrestrained mice were monitored daily. Baseline measurements of enhanced-pause (Penh) were recorded over 5 min and reported as an average. Lung function was measured daily prior to and following infection.

### Plaque assays

Whole lung and spleen homogenates were collected from MHV-1-infected C3H/HeJCr mice and viral titers were determined by plaque assay as previously described [17].

### Administration of clodronate liposomes

CL were obtained from Nico van Rooijen (Vrije University, Amsterdam, The Netherlands). Mice were depleted of AM by administering 75 µl of CL i.n. 6 hours (hr) prior to MHV-1 infection. Control mice were administered 75 µl of Dulbecco's phosphate buffered saline (PBS; Gibco, Grand Island, NY).

### Cell isolation from tissue

Bronchoalveolar lavage (BAL) cells were harvested by inserting a cannula into the trachea and performing 3 consecutive 1 ml washes with RPMI 1640 supplemented with 10 U/ml penicillin G, 10 µg/ml streptomycin sulfate, 2 mM l-glutamine, 0.1 mM nonessential amino acids, 1 mM sodium pyruvate, 10 mM HEPES (all from Gibco), 5×10^−5^ M 2-mercaptoethanol (Sigma-Aldrich, St. Louis, MO) and 10% fetal calf serum (FCS; Atlanta Biologicals, Lawrenceville, GA) into the lung airspace. Lungs were subsequently perfused by slowly injecting 5 mL of PBS into the right ventricle of the heart. The tissue was dissected and cut into small pieces and placed in 4 ml of HBSS w/o CaCl_2_, MgCl_2_ and MgSO_4_ (Gibco) supplemented with 125 U/ml Collagenase Type II (Invitrogen, Grand Island, NY) and 60 U/ml DNase I (Sigma-Aldrich). Lungs were incubated on a rotator at 37°C for 30 min. Lymph nodes were similarly digested in 1 ml of HBSS containing collagenase and DNase I as described above. Lungs were pressed through a wire mesh screen (Bellco glass, Vineland, NJ) to create a single-cell suspension. Lymph nodes and spleens were pressed between the frosted ends of glass slides to create single-cell suspensions (Surgipath, Richmond, IL).

### Immunophenotying

Single-cell suspensions of the various tissues were plated into 96-well round bottom plates at 1.5×10^6^ to 2×10^6^ cells/well. Cells were washed with staining buffer (PBS containing, 2% FCS and 0.02% sodium azide), blocked with anti-FcγRII/III monoclonal antibody (mAb; clone: 24G.2) and stained with fluorochrome-conjugated antibodies. The following antibodies were used: CD4-peridinin-chlorophyll proteins (PerCP; clone: RM4-5, Biolegend), CD49d-Alexa Fluor 647 (clone: R1-2, Biolegend), CD8-PerCP-Cy5.5 (clone: 53-6.7, Biolegend), CD11a-phycoerythrin (PE; clone: M17/4, eBioscience) and CD90.2- fluorescein isothiocyanate (FITC; clone: 53-2.1, eBioscience). Samples were acquired on a FACSCanto flow cytometer (Becton Dickinson, San Jose, CA) and analyzed using FlowJo software (Tree Star, Inc., Ashland, OR).

### Intracellular-cytokine staining

Cells were stimulated for 5 hr at 37°C in RPMI 1640 with 10 µg/ml of brefeldin A (BFA) alone or BFA with MHV-1 derived peptides. MHV-1-specific CD4 T cell responses were assessed using a pool of known CD4 T cell epitopes at a final concentration of 10 µg/ml: M196–210, N346–360, N376–390, S171–185, S881–895 and S921–935. MHV-1-specific CD8 T cell responses were assessed following stimulation with 1 µM of the N421–428 peptide. Cells were washed with staining buffer and surface stained with either Thy1.2 FITC and CD8 PerCP-Cy5.5 or Thy1.2 and CD4 for 30 min at 4°C. Cells were washed twice with staining buffer and subsequently fixed and red blood cells simultaneously lysed with FACS Lysing Solution (BD Bioscience) for 10 min at room temperature. Cells were washed with permeabilization buffer and stained with IFN-γ allophycocyanin (APC; clone: XMG1.2, Biolegend) and TNF-α PE (clone: MP6-XT22, eBioscience) mAb for 30 min. Cells were washed again with permeabilization buffer followed by a wash with staining buffer. In some cases cells were also stained with Foxp3 (clone: FJK-16s, eBioscience) using the Foxp3 Staining Kit (eBioscience) per the manufacturer's instructions. Samples were run on a FACSCanto flow cytometer (BD Bioscience) and data analyzed using FlowJo software.

### Statistical analysis

All statistical analyses were performed using Prism software (GraphPad Software, Inc., San Diego, CA). Statistical significance was determined by using an unpaired *t* test. A value of *p*<0.05 was considered significant. Asterisks indicating significance were defined as follows: *, *p*<0.05; **, *p*<0.01; ***, *p*<0.001.

## Results

### AM depletion following intranasal administration of CL

**Figure 1:**
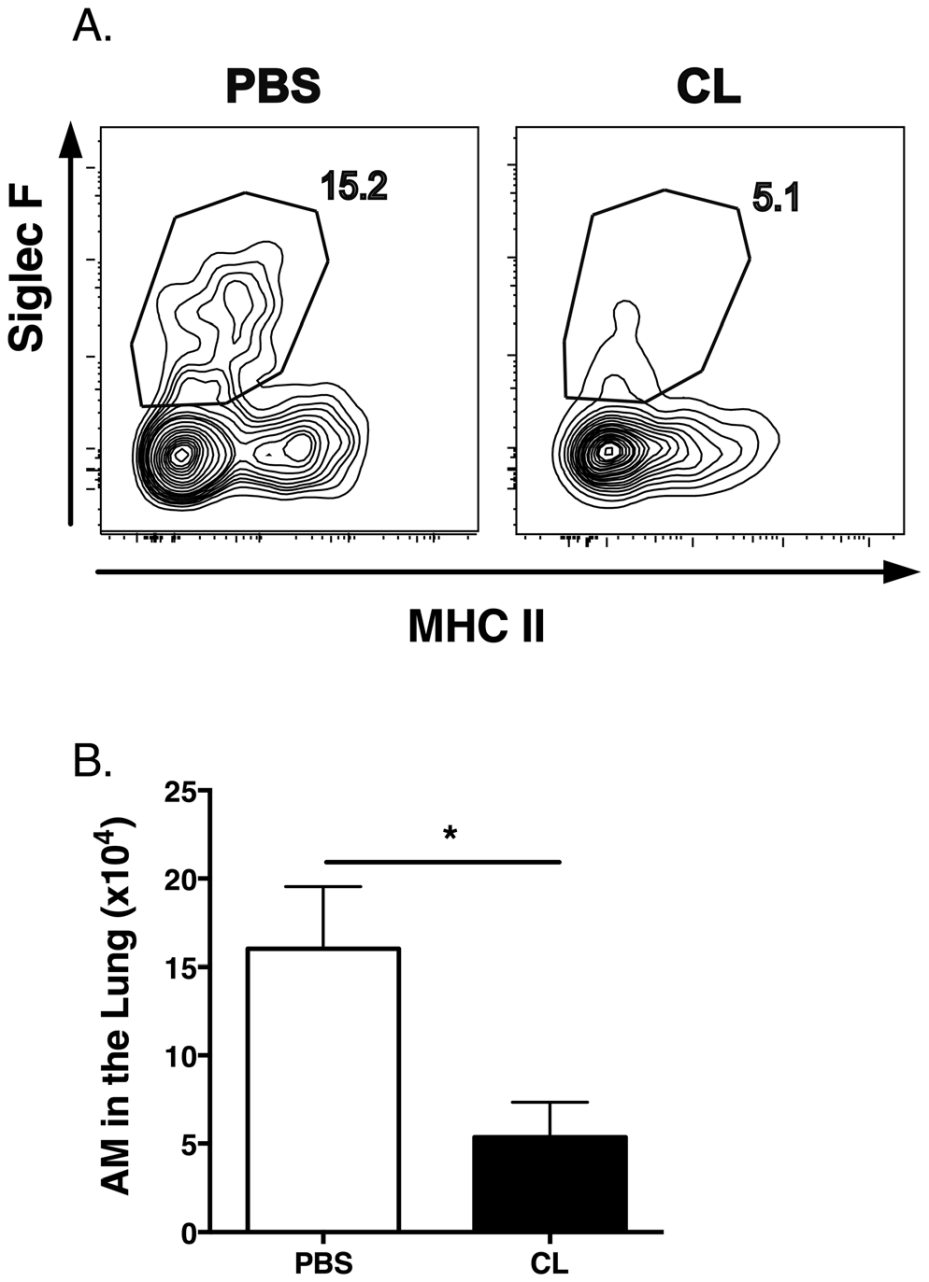
Sustained depletion of AM in the lung following CL administration at 48 hours. C3H/HeJ mice were administered 75 µl of either CL or PBS i.n. and lung mononuclear cells were isolated 48 hr later. Cells were stained for CD11c, CD11b, MHC class II and Siglec F. (A) Plots show Siglec F and MHC class II expression on gated CD11c^hi^/CD11b^lo^ AM. (B) Total AM numbers in the lung. Combined data from two individual experiments with *n* = 8/group is shown.

To verify that CL treatment would result in the selective depletion of AM within the lung, we examined the frequency and total number of AM in the lung at 6, 24 and 48 hr following i.n. CL administration. Depletion of AM defined as, CD11b^lo^/CD11c^hi^/MHCII^lo^/Siglec F^+^, was observed at 6 and 24 hr (data not shown) and was maintained for at least 48 hr (Figure 1A, B). The total number of AM in the lung at 48 hr was significantly (*p*<0.05) reduced as compared to PBS controls. Other pulmonary cell populations were unaffected at all time points examined (data not shown).

### AM Depletion reduces MHV-1-induced morbidity and mortality

**Figure 2:**
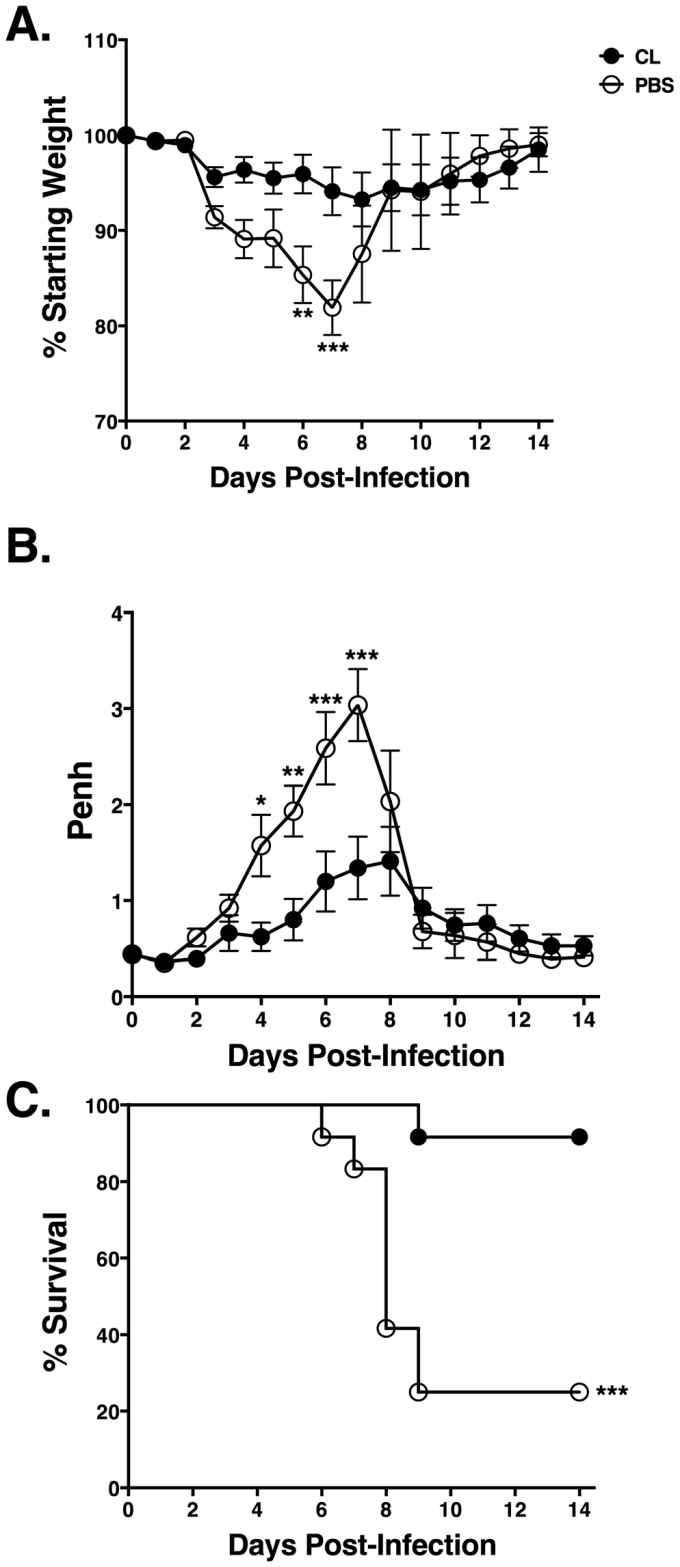
AM depletion reduces MHV-1-induced morbidity and mortality. Mice were treated with either 75 µl of CL or PBS 6 hr prior to MHV-1 infection. (A) Weight was monitored over the course of infection. (B) Penh was measured using a whole-body plethysmograph. (C) Kaplan-Meier Survival curves depict the frequency of surviving mice in each group at the indicated time points following infection. Combined data from three independent experiments is shown with *n* = 12 mice/group.

In order to evaluate the role of AM during respiratory MHV-1 infection, AM were depleted from C3H/HeJ mice via i.n. administration of 75 µl of CL 6 hr prior to infection. C3H/HeJ mice administered CL exhibited significantly less weight loss between days 6–7 post-infection (p.i.) as compared to PBS-treated control mice (Figure 2A). Similarly, CL-treated mice showed significantly reduced airway resistance (Penh) between days 4–7 p.i. as compared to the PBS control mice following MHV-1 infection (Figure 2B). Consistent with the reduced weight loss and improved airway function, the CL-treated mice also exhibited significantly increased survival (Figure 2C). The timing of AM depletion was critical as mice administered CL 48 hr after MHV-1 infection exhibited no difference in weight loss, Penh, or survival as compared to PBS control mice (data not shown). Thus, depletion of AM prior to MHV-1 infection results in significantly reduced morbidity and mortality.

### Impact of AM depletion on virus replication

**Figure 3:**
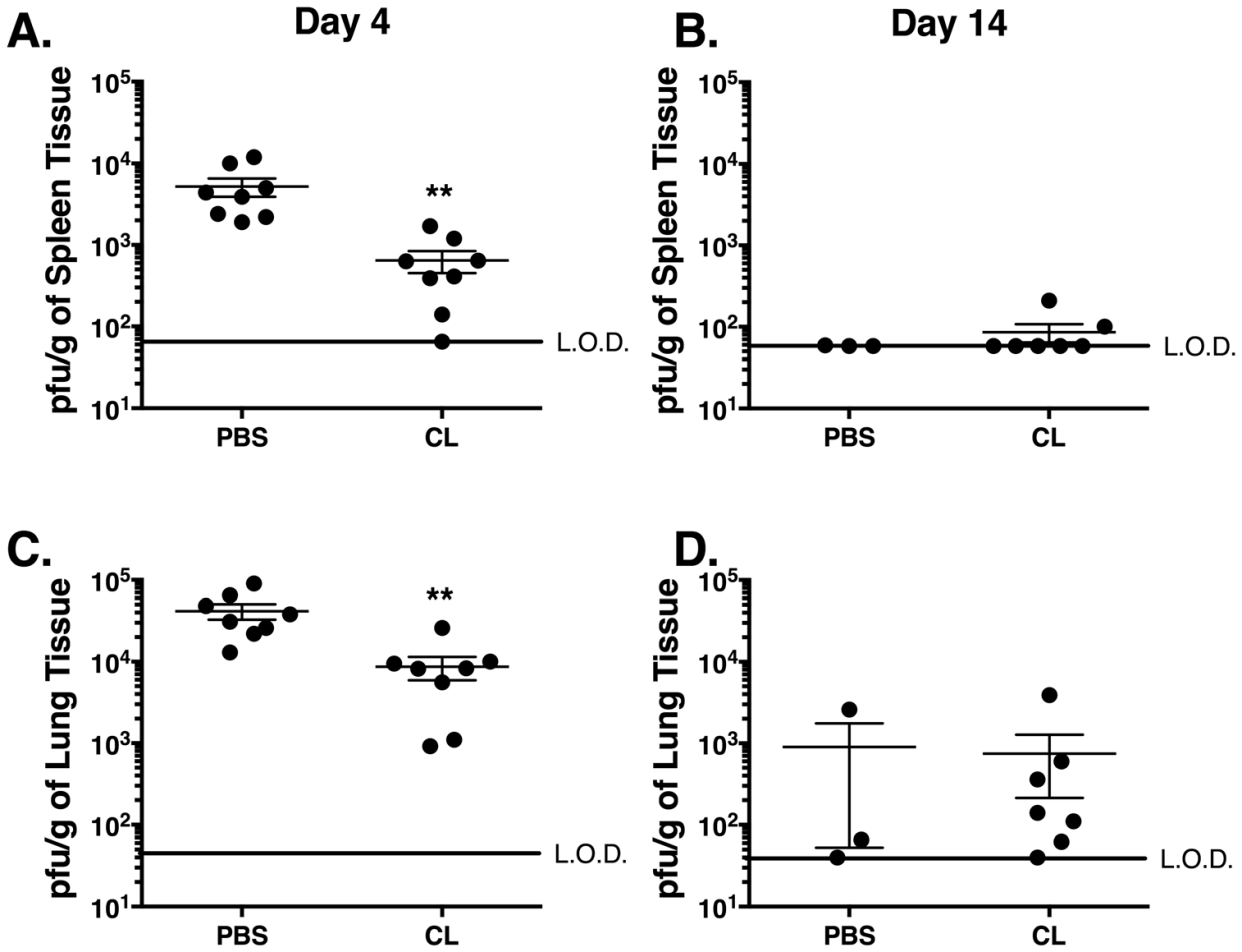
AM depletion results in reduced peak viral titers. Mice were treated with either 75 µl of CL or PBS 6 hr prior to MHV-1 infection. MHV-1 titers were determined in the (A, B) spleen and (C, D) lungs on days 4 and 14 p.i.. The mean ± SEM for each group is indicated by a short horizontal line. The limit of detection (LOD) is indicated on each graph by a long horizontal line. Combined data from two independent experiments is shown with *n* = 8 mice/group.

To determine the impact of CL administration on virus replication, viral titers were determined in the spleen and lung at days 4 and 14 p.i.. At day 4 p.i., the CL-treated mice exhibited significantly (*p*<0.01) reduced viral titers in the spleen and lung as compared to the PBS controls (Figure 3A, C). However, by day 7 p.i., viral titers were not significantly different between the groups (data not shown). At day 14 p.i., surviving mice from both groups had either cleared the virus from the spleen (Figure 3B) or exhibited similarly low levels of virus in the lung (Figure 3D). Thus, depletion of AM was associated with a decreased viral load early after infection.

### Impact of the AM depletion on the T cell response

**Figure 4:**
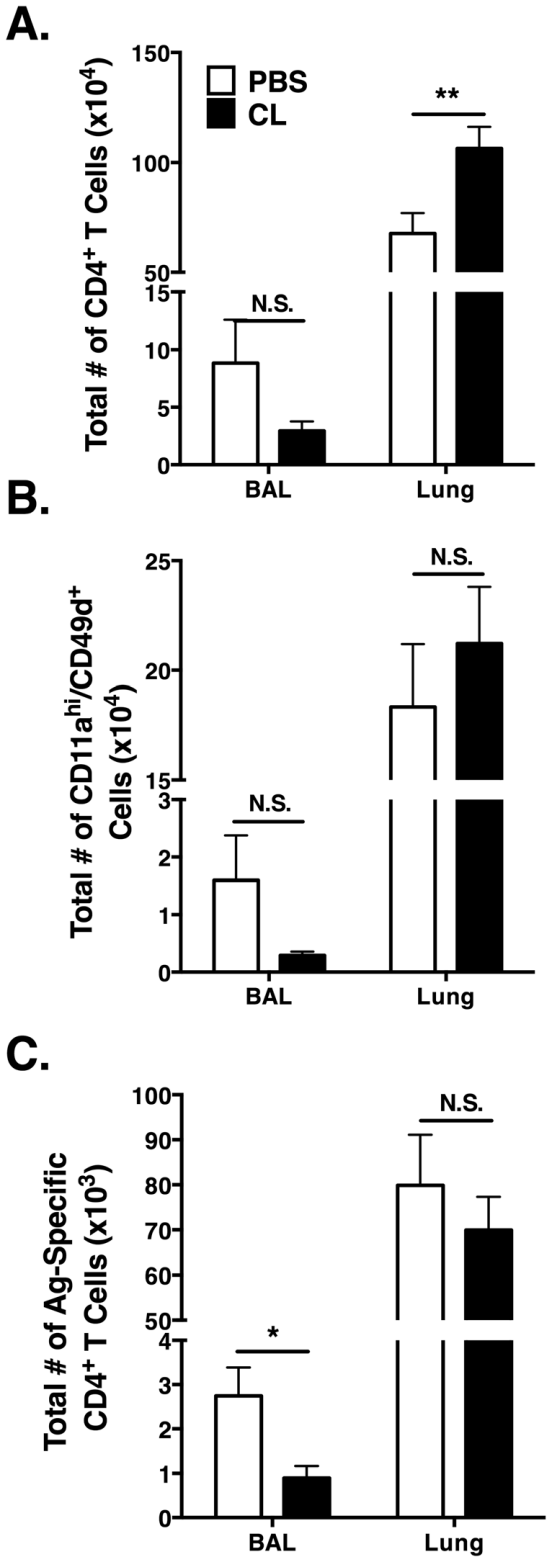
Impact of AM depletion on the CD4 T cell response in the lung following MHV-1 infection. Mice were administered 75 µl of either CL or PBS, 6 hr prior to MHV-1 infection. Bronchoalveolar lavage (BAL) cells from the lung airways and mononuclear cells from the lung tissue were isolated at day 6 p.i.. (A) Total number of CD4^+^ CD90.2^+^ T cells. (B) Total number of CD11a^hi^CD49d^+^ CD4^+^ T cells. (C) Total number of MHV-1-specific IFN-**γ** ^+^ CD4^+^ T cells by peptide stimulation from the following six MHV-1 peptides: M196–210, N346–360, N376–390, S171–185, S881–895 and S921–935. Combined data from five individual experiments in (A) and (B) with *n* = 20 mice/group. Combined data from three individual experiments in (C) with *n* = 12 mice/group.

We have previously reported that both CD4 and CD8 T cells contribute to MHV-1-induced morbidity and mortality [18]. Therefore we examined the impact of AM depletion on the MHV-1-induced T cell response. At day 6 p.i., prior to the onset of MHV-1-induced mortality, there was no significant difference between the CL-treated mice and the PBS control mice in total CD4 T cell numbers in the BAL (Figure 4A). However, we observed a significant (*p*<0.01) increase in the total number of CD4 T cells in the lung tissue of CL-treated mice as compared to the PBS control mice (Figure 4A). Using CD11a and CD49d expression to identify antigen-specific CD4 T cells [20], we observed no significant difference in the total number of CD11a^hi^/CD49d^+^ CD4 T cells in either the BAL or lungs of CL-treated mice as compared to PBS control mice (Figure 4B).

We have previously identified six MHV-1-derived CD4 T cell epitopes in C3H mice [21]. Because the frequency of the responding CD4 T cells to any one of these epitopes is relatively low, we used a pool of all six epitopes to stimulate the CD4 T cells ex vivo. We observed a significant (*p*<0.05) decrease in the total number of MHV-1-specific IFN-**γ**-producing CD4 T cells in the BAL of CL-treated mice as compared to PBS controls (Figure 4C). In addition, there was a slight decrease in the total number of IFN-**γ**-producing CD4 T cells in the lungs of CL-treated mice as compared to the PBS controls, though it did not reach significance (Figure 4C).

**Figure 5:**
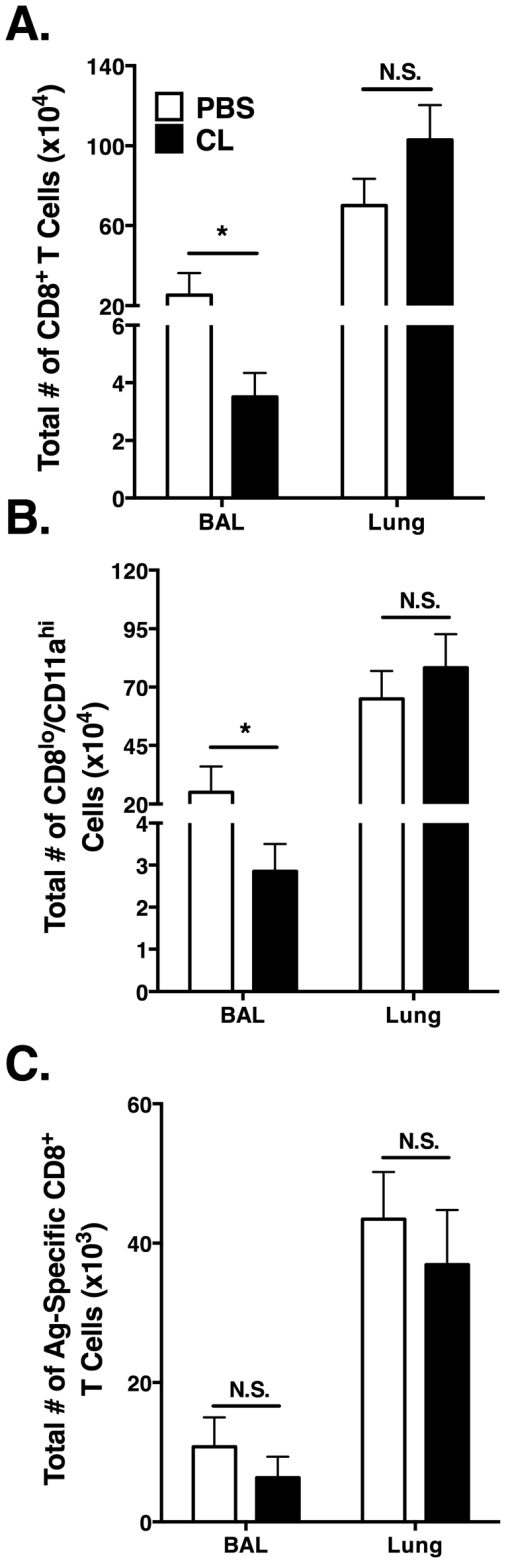
Impact of AM depletion on the CD8 T cell response in the lung following MHV-1 infection. Mice were administered 75 µl of either CL or PBS, 6 hr prior to MHV-1 infection. BAL cells from the lung airways and mononuclear cells from the lung tissue were isolated at day 6 p.i.. (A) Total number of CD8^+^ CD90.2^+^ T cells. (B) Total number of CD11a^hi^CD8^lo^ T cells. (C) MHV-1 antigen (Ag)-specific IFN-**γ** ^+^ producing CD8^+^ T cells were identified by N421-428 peptide stimulation. Combined data from five individual experiments in (A) and (B) with *n* = 20 mice/group. Combined data from two individual experiments in (C) with *n* = 8 mice/group.

We also evaluated the CD8 T cell response. At day 6 p.i., we observed a significant (*p*<0.05) decrease in the total number of CD8 T cells in the BAL of CL-treated mice as compared to the PBS control mice (Figure 5A). In contrast, the total number of CD8 T cells in the lung tissue was similar between the groups. Using surrogate markers to identify antigen-specific CD8 T cells [22], [23], we observed a significant (*p*<0.05) decrease in the total number of CD8^lo^/CD11a^hi^ CD8 T cells in the BAL of CL-treated mice as compared to the PBS control mice (Figure 5B). However, we observed no difference in the total number of CD8^lo^/CD11a^hi^ CD8 T cells in the lungs of CL-treated mice compared to the PBS control mice (Figure 5B).

In order to evaluate the virus-specific CD8 T cell response, we stimulated cells isolated from the lung with the D^k^-restricted N421-428 epitope [21]. The total number of MHV-1-specific IFN-**γ**producing cells in the BAL and the lungs was slightly reduced in mice administered CL as compared to the PBS control mice, however this reduction did not reach significance (Figure 5C).

### Increase in Tregs following AM depletion

**Figure 6:**
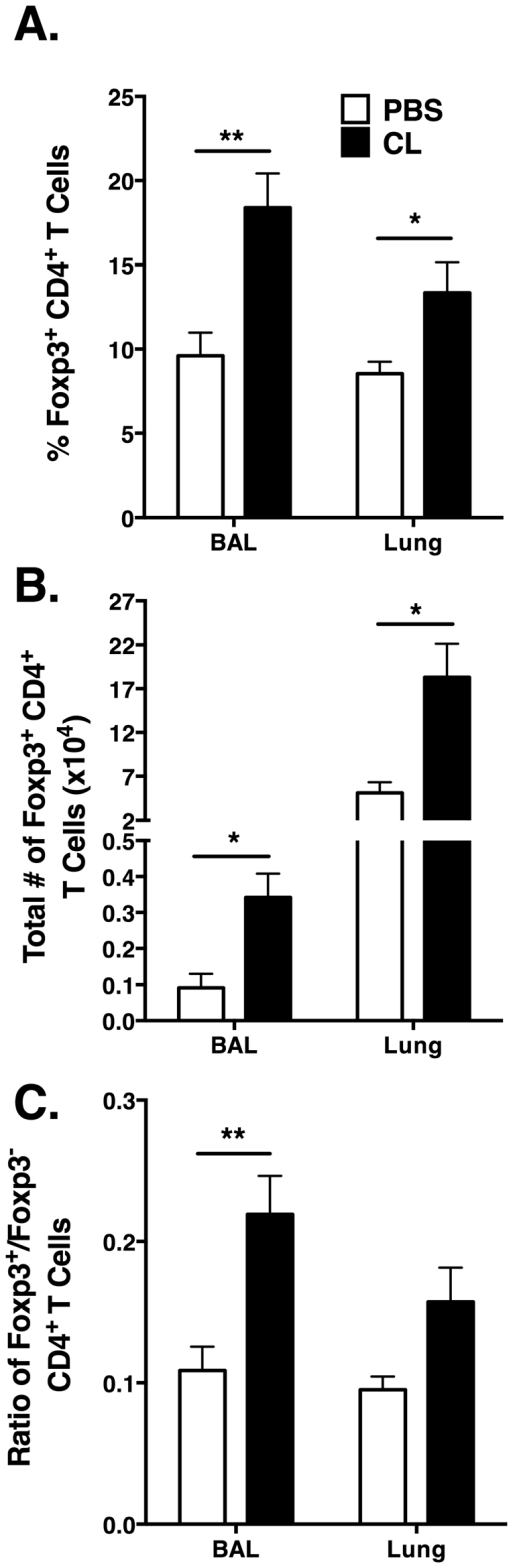
AM depletion results in an increased number of Foxp3^+^ Tregs in the lung following MHV-1 infection. Mice were administered 75 µl of either CL or PBS, 6 hr prior to MHV-1 infection. BAL cells from the lung airways and mononuclear cells from the lung tissue were isolated at day 6 p.i.. (A) Frequency of Foxp3^+^ CD4 T cells. (B) Total number of Foxp3^+^ CD4 T cells. (C) The ratio of Foxp3^+^ Tregs to Foxp3^−^ CD4 T cells was determined in the BAL and lungs of CL-treated mice as compared to control mice. Combined data from two individual experiments with *n* = 8 mice/group.

Regulatory T cells (Tregs) are known to suppress T cell responses [24]. Because our data indicated a general trend of reduced CD4 and CD8 T cell responses in the lung airways, we hypothesized that AM depletion may have altered the Treg response. We had noted earlier that the total number of CD4 T cells in the lungs of CL-treated mice is increased, but there was not a similar increase in the number of the virus-specific CD4 T cells. Therefore, we questioned if an increase in Foxp3^+^ Tregs would account for this apparent discrepancy. We observed significant increases in the both frequency and total number of Foxp3^+^ CD4 T cells in the BAL (*p*<0.01) and the lungs (*p*<0.05) of the CL-treated mice at day 6 p.i. as compared to that of the PBS control mice (Figure 6A, B). We also observed that the ratio of Foxp3^+^ CD4 T cells to Foxp3^−^ CD4 T cells was significantly increased in the BAL (*p*<0.01) and although it did not reach significance, the lung exhibited a similar trend (Figure 6C). Thus, depletion of AM was associated with a significant increase in the number of Tregs in the lung airways and lung tissue following MHV-1 infection.

## Discussion

Here we examined the impact of AM depletion via CL treatment on MHV-1-induced morbidity and mortality in susceptible C3H/HeJ mice. We have previously shown that C3H/HeJ mice are more susceptible than either BALB/c or C57BL/6 mice to an intranasal MHV-1 infection [17]. Our results indicate that MHV-1-induced morbidity and mortality is reduced in mice administered CL prior to infection as compared to PBS control mice. It is known that some strains of MHV can replicate within macrophages [25]. Therefore, it is possible that the protection we observe following AM depletion is due to the elimination of a population of potential viral target cells. This may explain why we observe a significant reduction in peak viral titers in CL-treated mice as compared to PBS controls. In contrast to our results, work by Luker *et al*. has shown that during a vaccinia virus infection, depletion of AM results in increased weight loss and viral replication [26]. Although vaccinia virus may also infect AM [26], in the study by Luker *et al*. CL were administered intratracheally (i.t.) 2 days prior to infection [26] as compared to i.n. administration 6 hr prior to infection as we have done here, which may account for the differences in outcomes.

AM are known to suppress the immune response [27]–[29]. Depletion of AM protects BALB/c mice from a lethal infection with a mouse-adapted strain of SARS-CoV [29]. Similar to our findings, SARS-CoV-infected mice quickly regain body weight. We demonstrate that depletion of AM prior to infection also results in reduced total numbers of CD4 and CD8 T cells in the BAL of CL-treated mice as compared to PBS control mice. We also observe reduction in the total number of antigen-specific CD4 and CD8 T cells in CL-treated mice as compared to PBS control mice. However, in contrast to our results, AM depletion resulted in an increase in the total and SARS-CoV-specific T cell response in the lung tissue [29]. The differences between our results and those detailed above could be explained by the timing of CL administration. In the SARS-CoV studies, CL was administered 3 days prior to SARS-CoV infection, which may have allowed for AM numbers to recover earlier during the course of infection.

Openshaw *et al*. demonstrated that BALB/c mice treated with CL 3 days prior to an acute respiratory syncytial virus (RSV) infection results in no significant changes in either CD4 or CD8 T cell recruitment or T cell activation [30]. In addition, morbidity and mortality of these mice were also unaffected. An increase in viral titers at the peak of infection was noted, but no significant differences were observed in viral clearance. Thus, it is clear that the timing of AM depletion and the viral system examined both contribute to the impact of AM depletion on a pulmonary viral infection.

A recent study by Soroosh *et al*. explored the relationship between AM and the development of Foxp3^+^ inducible Tregs (iTregs) in the lung. CD11c^+^ F4/80^+^ macrophages were found to promote the generation of Foxp3^+^ iTregs through the expression of TGF-β and retinoic acid under steady state conditions [31]. Following allergen exposure, the AM lost their capacity to promote iTreg generation and converted to an inflammatory phenotype [31]. Work by Savage *et al*. also showed that Treg induction could be regulated by the activation state of AM. CD4 T cells exhibit a more functional suppressor phenotype after encounter with anti-inflammatory type 2 macrophages whereas pro-inflammatory type 1 macrophages promote proliferation in the presence of antigen [32]. These studies demonstrate the importance of AM and their role in controlling lung inflammation and influencing the generation of Tregs.

Tregs have been shown to play an important roll in modulating the adaptive immune response, as well as play a critical roll limiting immunopathology during an acute pulmonary virus infection, as seen with RSV [33] and influenza A virus (IAV) [34]. The frequency of Tregs in the lung has been reported to be increased following an infection with either RSV [33] or IAV [34]. We observed a significant increase in the total number of CD4 T cells in the lung tissue of CL-treated mice. We also observed a significant increase in the total number and frequency of Tregs following MHV-1 infection in both the airways and lung tissue of the CL-treated mice. The increased Treg response we observe may help modulate the pathogenic CD4 and CD8 T cell response resulting in reduced morbidity and mortality.

In this report we show that the depletion of AM by CL administration prior to a MHV-1 infection is associated with a significant amelioration of disease. Depletion of AM prior to MHV-1 infection significantly reduces weight loss and airway function and increases survival in susceptible C3H/HeJ mice. We have previously shown that both CD4 and CD8 T cells contribute to morbidity and mortality in the model [18]. Our results suggest that depletion of AM prior to MHV-1 infection modulates the development of a pathogenic T cell response.
